# miRNA Expression Profiling in Human Breast Cancer Diagnostics and Therapy

**DOI:** 10.3390/cimb45120595

**Published:** 2023-11-25

**Authors:** Iga Dziechciowska, Małgorzata Dąbrowska, Anna Mizielska, Natalia Pyra, Natalia Lisiak, Przemysław Kopczyński, Magdalena Jankowska-Wajda, Błażej Rubiś

**Affiliations:** 1 Department of Clinical Chemistry and Molecular Diagnostics, Poznan University of Medical Sciences, Rokietnicka 3, 60-806 Poznan, Poland; iga_dz@op.pl (I.D.); gosia.guciol@gmail.com (M.D.); amizielska@ump.edu.pl (A.M.);; 2 Centre for Orthodontic Mini-Implants, Department and Clinic of Maxillofacial Orthopedics and Orthodontics, Poznan University of Medical Sciences, Bukowska 70 Str., 60-812 Poznan, Poland; 3 Faculty of Chemistry, Adam Mickiewicz University, Uniwersytetu Poznanskiego 8 Str., 61-614 Poznan, Poland; magdajw@amu.edu.pl

**Keywords:** breast cancer, miRNA profiling, cancer diagnostics, cancer therapy

## Abstract

Breast cancer is one of the most commonly diagnosed cancer types worldwide. Regarding molecular characteristics and classification, it is a heterogeneous disease, which makes it more challenging to diagnose. As is commonly known, early detection plays a pivotal role in decreasing mortality and providing a better prognosis for all patients. Different treatment strategies can be adjusted based on tumor progression and molecular characteristics, including personalized therapies. However, dealing with resistance to drugs and recurrence is a challenge. The therapeutic options are limited and can still lead to poor clinical outcomes. This review aims to shed light on the current perspective on the role of miRNAs in breast cancer diagnostics, characteristics, and prognosis. We discuss the potential role of selected non-coding RNAs most commonly associated with breast cancer. These include miR-21, miR-106a, miR-155, miR-141, let-7c, miR-335, miR-126, miR-199a, miR-101, and miR-9, which are perceived as potential biomarkers in breast cancer prognosis, diagnostics, and treatment response monitoring. As miRNAs differ in expression levels in different types of cancer, they may provide novel cancer therapy strategies. However, some limitations regarding dynamic alterations, tissue-specific profiles, and detection methods must also be raised.

## 1. Introduction

Breast cancer (BC) is the most commonly diagnosed cancer worldwide [1,2]. This disease constitutes approximately 11.7% of total cancer cases. It has recently overtaken lung cancer, which is assessed at 11.4%. Consequently, breast carcinoma is the leading cause of death among females and the fifth among both sexes (although rare in males) [3,4,5]. Non-invasive breast cancer ductal and lobular carcinomas in situ represent 15% of total breast malignancies. The first type develops in milk ducts, whereas the latter originates in breast lobules. However, in both cases, cells can transform and become invasive [6].

Without a doubt, breast malignancy is heterogeneous, which makes it more challenging to diagnose. The entities of neoplasms differ regarding how they were triggered, how they respond to therapy, and the outcome. According to the World Health Organization (WHO), there are at least 18 histological types of breast carcinoma. There is a significant difference between histological and molecular classification. Luminal, HER2-enriched (human epidermal growth factor receptor 2), basal-like, and normal breast-like are identified as four molecular subtypes. The luminal is further divided into subgroups: luminal A and luminal B [7]. The molecular taxonomy involves profiling of gene expression, evaluated at the mRNA levels, which provides a high sensitivity of detection. Early detection plays a crucial role in decreasing mortality and providing a better prognosis for patients.

Breast malignancy has numerous risk factors such as sex, age, having children, age at first birth, family history, genetic background, taking birth control pills, alcohol consumption, smoking, etc. [4,8]. The prognosis and treatment of breast carcinoma depends, among other things, on tumor-node-metastasis staging. The other crucial factors are lymphovascular spread, age and menopausal status of the patient, histological grade, hormone receptor status, and overexpression of ERBB2/HER2 (erythroblastic oncogene B2/HER2) [9]. Altogether, dealing with unequivocal diagnostics, metastasis, resistance to drugs, and recurrence are the burdens of breast cancer treatment that result in severe limitations in therapy efficacy. That is why developing novel diagnostics and treatment strategies is so valid. It seems that an epigenetics assessment covering micro-ribonucleic acid (miRNA) profiling might significantly contribute to better patient outcomes as it refers to many pathways involving oncogenes or tumor suppressor genes [10,11,12].

## 2. Breast Cancer Treatment Strategies Based on Molecular Characteristics

Early cancer diagnosis increases treatment options and patients’ survival. Available diagnostic strategies are based on medical imaging and biomarker analysis. In 20 to 30% of invasive breast cancer cases, the overexpression or amplification of HER2 is observed [13,14,15]. Dimerization of the receptor initiates various signaling pathways, leading to cell proliferation and tumorigenesis. This is why HER2-positive cells are more aggressive than HER2-negative breast cancer cells [16]. There are some known upregulated miRNAs in HER2+ breast cancer patients, e.g., miR-4728 or miR-146a-5p, and also there are miRNAs which are HER2 cell signaling inhibitors, like miR-33b, miR-491-5p, miR-634, and miR-637 [17,18,19,20]. Furthermore, miR-342-5p and miR-744 are significantly downregulated in HER2-positive breast tumors as compared to HER2-negative tumors, and higher expression of miR-342-5p is associated with better survival in both HER2-positive and HER2-negative breast cancer patients [17].

Targeted therapy for HER2-positive breast cancer uses monoclonal antibodies such as trastuzumab (known as Herceptin), pertuzumab, and margetuximab [16]. They bind HER2 protein, attenuate proliferation signaling, and decrease cancer growth. This protein is a kinase that can also be blocked with drugs such as lapatinib or neratinib, which are kinase inhibitors [16]. Alternatively, another strategy can be involved that is based on an antibody–drug conjugate (ADC) [16]. One of the examples is ado-trastuzumab emtansine (Kadcyla), a combination of Herceptin and the chemotherapeutic drug emtansine [16]. It is used to treat early-stage breast cancer after surgery or in advanced stage after chemotherapy. The FDA also approved Enhertu (a HER2-directed antibody and topoisomerase inhibitor conjugate) that works for patients with an inoperable or metastasized tumor [16]. Another strategy is based on targeting hormone receptors (in estrogen-positive cancers). Cancer can also be targeted with drugs such as CDK4/6 inhibitors (palbociclib, ribociclib, and abemaciclib) that enable the slowing down of cancer development. The drugs block cyclin-dependent kinases (CDKs) and stop cells from dividing [18]. Similarly, some well-known mTOR inhibitors (e.g., sirolimus, everolimus, and temsirolimus) attenuate the malignancy potential of cancer cells [19]. Another signaling pathway effectively blocked in cancer is the PI3K pathway (found to control the proliferation and survival of breast cancer that results in tumor growth inhibition) [16].

However, one of the most critical pathways in cancer development is associated with BRCA genes (BRCA 1 and BRCA 2), which are related to DNA repair and cell cycle control [20]. The human BRCA1 mRNA 3′UTR region is predicted to bind 20–100 miRNAs, whereas some of these, e.g., miR-146a, miR-146b-5p, miR-182, miR-16, miR-17, miR-15a, and miR-638, were shown to regulate BRCA1 expression [21]. BRCA1 epigenetically represses miR-155, and overexpression of miR-155 accelerates tumor cell line growth in vitro [22]. Moreover, there are also miRNAs such as miR-155, miR-148, miR-152, miR-205, miR-99b, and miR-146a, which are targeted by BRCA1 [21]. BRCA1 was shown to be associated with the expressions of both precursor and mature forms of let-7a-1, miR-16-1, miR-145, and miR-34a [23].

The risk of developing breast (or ovarian) cancer in carriers of mutations in these tumor suppressor genes is significantly higher than in non-carriers. Precisely, in the general population, about 13% of women will eventually develop breast cancer [24], while 55–72% of women who inherit a harmful BRCA1 variant and 45–69% of women who inherit a harmful BRCA2 variant will develop breast cancer by 70–80 years of age [25,26,27]. Noteworthy, the risk depends on several factors, some of which have not been fully characterized. Another protein associated with the DNA repair pathway is PARP (poly (ADP-ribose) polymerase), which can be targeted with olaparib and talazoparib [16,20]. However, these strategies are not efficient enough in diagnostics (mutation detection) or therapy. Thus, providing novel diagnostic and therapeutic targets is still highly important, and miRNA could be a promising area.

## 3. miRNAs—The Mechanism of Action

miRNAs are defined as endogenous, 21–25 nucleotide single-stranded RNAs (ssRNAs) that are produced from hairpin-shaped precursors [25]. These molecules are involved in processes crucial for development and general metabolism. The role of these non-coding RNAs covers cell proliferation, differentiation, apoptosis, and tumorigenesis [26]. As shown in *Drosophila melanogaster*, miRNAs controlled cell death, proliferation, and Notch signaling [27,28,29]. In mice, they were shown to contribute to T-cell development and innate immunity [30,31,32]. In humans, miRNAs were shown to participate in the regulation of granulocyte maturation [33], development and function of the immune system [34], adipocyte differentiation [35], antiviral defense [36], gene downregulation in colon adenocarcinoma [37] and upregulation in B-cell lymphoma [38,39], and many other functions. Recent studies have shown the pivotal role of specific miRNAs in the development, progression, and cancer response to treatment [40,41,42]. It was also suggested that miRNAs could function as breast cancer biomarkers due to their aberrant expression [7].

The miRNA-related mechanism of gene expression modulation is related to the post-transcriptional effect, possibly due to the base pair complementarity with mRNA molecules. The gene silencing process can be conducted with mRNA cleavage or inhibition of transcript translation [43]. Predominantly, miRNAs bind to the sequence at the 3′ UTR of their target mRNAs, but binding to other mRNA regions such as the 5′ UTR and the coding sequence was also revealed [43]. Significantly, miRNA binding to 3′ UTR and coding regions contributes to gene expression silencing, while binding the promoter region results in transcription induction [43]. miRNA does not usually show complete complementarity to the 3′ UTR, which enables the targeting of many of genes, some of which may be involved in carcinogenesis [44]. The miRNA genes are situated directly at or near mutation-prone sites of chromosomes. Thus, DNA damage influences the expression of tumor suppressors and miRNAs. The variety of miRNA expression that regulates cancer-related genes make miRNAs a new class of oncogenes and tumor suppressor genes [26].

**Figure 1:**
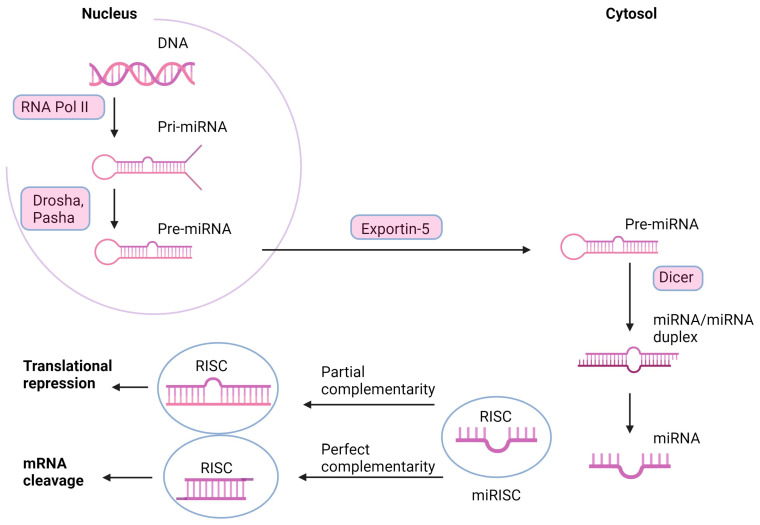
The transcription and mechanism of miRNA action (according to [45], created in BioRender.com). The microRNA gene is operated by RNA polymerase II (Pol II) into primary microRNA (pri-miRNA), which is prepared into pre-miRNA hairpins by Drosha and Pasha (RNase III enzyme and its cofactor). Then, the pri-miRNA is exported by Exportin-5 from the nucleus to the cytoplasm, where Dicer cleaves it by the ribonuclease (RNase) activity into a transient miRNA/miRNA duplex intermediate. The duplex loads onto components in the RNA-induced silencing complex (RISC), separate, and leads to double-stranded helix formation by complementing the antisense strand with the mRNA sequence target. mRNA could bind with complete complementarity, which leads to endonucleolytic cleavage, or just with partial complementarity, which drives translational repression, probably by a bulge sequence formation.

The miRNA mechanism of action and transcription starts in the nucleus, from miRNA genes. Formation of pri-miRNA is operated by RNA polymerase II (Pol II), Drosha and Pasha cofactor, into 60- to 110-nucleotide pre-miRNA hairpins. It is exported to the cytosol, where it is cleaved by the RNase activity of Dicer into a transient, 22-nucleotide miRNA/miRNA duplex intermediate. The duplex loads onto components in the RNA-induced silencing complex (RISC) and separates. Further, the miRNA-RISC complex leads to double-stranded helix formation by complementing the antisense strand with the mRNA sequence target. If mRNA is bound with complete complementarity, it encounters endonucleolytic cleavage (Figure 1). Partial complementarity leads to translational repression, probably by forming a bulge sequence in the middle of the helix [45].

## 4. The Potential of miRNAs in Current Cancer Diagnostics and Therapy

By targeting numerous transcripts, miRNAs affect pathways, leading to different phenotypic status [46]. Over the years, abnormal levels of various miRNAs have been reported in many cancers, including breast cancer. There is a strong belief that miRNA expression profiles could become predictive and prognostic biomarkers, similar to protein-coding gene expression assessment. The levels of individual miRNAs differ between breast cancer stages, which could also be used in future diagnostics. Depending on the type of cells, the same miRNAs can show oncogenic or tumor suppressor properties [47,48,49].

As reported, miRNAs interact with cell signaling pathways and affect breast cancer metastasis and progression. They can regulate TGF-β (miR-106b, miR-200 family, miR-106b; the effects are mainly mediated by the downregulation of SMAD), Wnt/β-catenin (miR-4469 is a main inducer of the pathway in breast cancer cells while miR-34a is the main repressor), NF-κB (directly targeted by miR-29a, which controls proliferation, cell cycle, and apoptosis, but also controlled by miR-1246, miR-449a, or miR-200b), PI3K/Akt/mTOR (miR-21 is the main inducer of the pathway), Notch (miR-34a plays a critical function in sustaining breast cancer stem cells), and ERK/MAPK (controlled by miR-543, miR-200c, or miR-148a/152) pathways [50]. Significantly, the latter can contribute to many pathways related to the differentiation and migration of breast cancer cells. Various studies showed a correlation between individual miRNAs and ERK/MAPK and the ability of miRNAs to downregulate or upregulate this pathway [50]. For example, miR-543 was shown to impair tumor growth and proliferation in breast cancer cell lines (MCF-7, MDA-MB-231, MDA-MB-453, HCC-1937) by inhibiting ERK2 activity [51]. Noteworthy, abnormal activation of receptor tyrosine kinases (RTKs, one of the mediators in the ERK/MAPK pathway) results in the progression of various cancers. This pathway (also known as the Ras-Raf-MEK-ERK pathway) conveys extracellular information to the DNA in the nucleus and takes part in cell proliferation and differentiation control [51,52]. The pathway can be initiated with growth factors, cytokines, or hormones. They bind to the two subunits of a RTK, followed by the dimer formation. RTK binds to adaptor proteins, which attract guanine–nucleotide exchange factors (GEFs). These factors displace GDP from RAF proteins and allow GTP to bind, which causes RAS activation. Then, further protein kinases, RAF, and MEK are activated. The final enzyme MAPK (ERK) is translocated to the nucleus and activates transcription factors [51,52,53]. RTK are transmembrane proteins that mediate cell-to-cell communication. The aberrant activation of RTKs is found as a cancer progression factor [52]. Thus, they are used as therapeutic targets. Unfortunately, tyrosine kinase inhibitors have multiple side effects, including diarrhea, nausea, vomiting, oral ulceration, headache, and dizziness [54]. In addition, the drug-resistance cases of tyrosine kinase inhibitors (TKI) are already known [54]. Therefore, the use of RTK inhibitors has become limited. Thus, novel, more specific strategies, e.g., miRNA-based strategies, are expected.

## 5. microRNA Profiling

miRNAs were shown to modulate the chemosensitivity of cancer cells to therapeutic agents, but this relationship is still unclear. Due to the diagnostic potential in breast cancer, miRNA profiling has become of interest in many studies. However, first, high-efficacy isolation must be provided. miRNA isolation can be performed from various biological samples such as cells in culture, tissue, blood plasma, serum, and other body fluids. miRNAs are more stable than mRNAs in blood plasma and serum, contributing to their potential use in gene regulation research. There are some challenges in selecting a method for miRNA profiling. microRNAs represent a small part of the total RNA fraction and can differ by a single nucleotide, which makes them more challenging to identify and distinguish. Thus, it is crucial to select the appropriate microRNA profiling method. Each method has its advantages, disadvantages, and limitations. They differ in the required amount of RNA, sensitivity, specificity, and costs.

One established method is real-time quantitative reverse transcription PCR (qRT-PCR), based on the reverse transcription of miRNA to cDNA and polymerase chain reaction (SybrGreen- or probe-labeled systems). Another method, microarray, relies on the hybridization of the labeled miRNA using capture probes, but this method cannot be used to determine absolute quantification. Another hybridization-based method is Nanostring nCounter, but one of the most modern methods is based on next-generation sequencing that allows the distinguishing of different miRNAs with very high accuracy [55,56,57,58]. Eventually, based on the collected literature data, several upregulated or downregulated miRNAs can be listed in breast cancer (Table 1). We consider them as potential cancer biomarkers that can become useful in medical diagnostics.

### 5.1. miR-21

In 2019, a study investigating miR-21 levels in the plasma of breast cancer patients and breast cancer cell lines was published. The study included 127 healthy patients (controls), 82 patients with benign breast tumors, and 252 with breast cancer. The levels of miR-21 were found to be different between these groups—the lowest miR-21 levels were found in healthy controls, while an increase in miR-21 levels was observed in patients with breast cancer. miR-21 levels were also compared between patients with different stages of the disease. Plasma miR-21 levels of breast cancer patients were correlated with the tumor, node, and metastasis (TNM) stage. In particular, an increase in miR-21 level was observed in the T3 stage, meaning the tumor is bigger than 5 cm. Then, the breast cancer cell lines Hs578T and MDA-MB-231 were transfected with a miR-21 inhibitor. After 14 days, it was found that colony formation ability was reduced in transfected cells compared with the controls. Transwell and wound healing tests were performed using the same cell lines to assess the ability of the cells to migrate. The tests confirmed that the miR-21 inhibitor reduced cell migration capacity. These results showed that inhibition of miR-21 could reduce metastasis and breast cancer proliferation. This means that therapies with miR-21 inhibitors might constitute a promising strategy for breast cancer patients [100,101]. miR-21 was also shown to play a crucial role in regulating drug resistance in breast cancer, and its overexpression was correlated with the development of multidrug resistance (MDR) [58]. Specifically, the research investigating the association of miR-21 expression with drug resistance in breast cancer indicated that miR-21 modulated the resistance of breast cancer cells to doxorubicin [58]. The study used a breast cancer cell line (MCF-7) and a doxorubicin-resistant breast cancer cell line (MCF-7/ADR). As reported, miR-21 expression was increased in MCF-7/ADR cells relative to MCF-7 cells. Importantly, one of the targets for miR-21 is the tumor suppressor gene PTEN, and in this study, PTEN expression was downregulated in MCF-7/ADR cells. This study suggested that miR-21 overexpression was associated with doxorubicin resistance to breast cancer and mediated by targeting phosphatase and tensin homolog (PTEN) [58,61]. Similarly, other research showed that miR-21 targeted insulin-like growth factor binding protein 3 (IGFBP3), which can be associated with brain metastases of BC cells. miR-21 was shown to cause an increase in cancer cell proliferation, migration, and the epithelial-to-mesenchymal transition (EMT) mediated by targeting TPM1, PCD4, and TGF-beta1 [64]. Knockdown of this particular miRNA was reported to induce cell apoptosis and inhibit proliferation and invasion of EMT [64].

### 5.2. miR-106a

It was reported that miR-106a was overexpressed in breast cancer tissue compared with normal tissue and was correlated with enhanced breast cancer cell proliferation. It was also associated with the downregulation of P53, BAX, and RUNX3 and the upregulation of Bcl-2 and ABCG2, which promote breast cancer cell proliferation. In addition, it was reported that upregulation of miR-106a decreased cell sensitivity to cisplatin [69]. The study in the mouse model also showed that miR-106a overexpression affected drug chemosensitivity. MDA-MB-231 and MCF-7 cell lines were treated with miR-106a inhibitor and miR-106a mimic. The mice were used to make a transplanted tumor model, and then the cisplatin treatment was added. The inhibition of tumor growth was observed when the inhibitor was applied, suggesting an association between miR106a and tumor sensitivity to cisplatin [102]. Altogether, it was reported that miR-106a could contribute to enhanced cell proliferation due to lowered sensitivity to chemotherapeutic agents [69,102]. Another study revealed a possible correlation between miR-106a levels and breast cancer cell proliferation mediated by RAF-1, activating the MAPK/ERK signaling pathway [68].

### 5.3. miR-155

miR-155 is an oncogenic miRNA involved in breast cancer growth regulation and is upregulated in breast cancer specimens. It was shown to contribute to telomere destabilization due to targeting TRF1 (shelterin component) [74] in MCF-7, SK-BR-3, and MDA-MB-468 cells. miR-155 overexpression was reported to reduce the expression of TRF1, leading to increased chromosome instability. Interestingly, reducing miR-155 levels showed an opposite effect [73,74,103,104].

### 5.4. miR-141

It was reported that the levels of miR-141 were decreased in breast cancer cells relative to surrounding tissues (qPCR) [78]. Additionally, the miR-141 level was correlated with the tumor stage. As demonstrated, overexpression of this miRNA was associated with decreased cell proliferation and enhanced apoptosis. Moreover, wound healing, assays, showed that miR-141 overexpression was accompanied by the inhibition of MDA-MB-231 cell migration. More detailed analysis revealed that one of the miR-141 targets, acidic nuclear phosphoprotein 32 family member E (ANP32E), was manifested by a significantly decreased level of ANP32E both at mRNA and protein levels in the miR-141 mimics transfected group [78]. Consequently, experiments with specific vshRNAs revealed that ANP32E knockdown inhibited MDA-MB-231 cell proliferation [78]. The relationship between ANP32E and triple-negative breast cancer was studied and it was demonstrated that ANP32E promotes tumor proliferation and the G1/S transition [79].

Another study demonstrated that miR-141-3p overexpression was correlated with aggressive breast carcinoma cases. The miRNA expression was compared between different breast tissues (malignant and benign), and significantly high miR-141-3p expression was demonstrated in grade III breast cancer compared to grade II [105]. These results suggested that miR-141-3p could discriminate malignant from benign breast tissues and, even more, could distinguish TNBC (triple-negative breast cancer) from other molecular subtypes of breast cancer. Altogether, miR-141-3p expression was correlated with shorter overall patient survival [105]. Additionally, assessment of the combination of miR-141-3p, miR-181b1-5p, and miR-23b-3p was suggested as a useful approach in cancer molecular subtypes identification.

### 5.5. Let-7c miRNA

The let-7 family of microRNAs are known to act as tumor suppressors [81]. Specifically, the let-7c level in the breast cancer patients’ serum and tissues was lower than in the controls. The association between let-7c expression levels and ER/PR status was investigated, but no significant difference was detected [81]. Interestingly, the upregulation of let-7c in premenopausal patients compared with postmenopausal patients was shown [81]. Another study suggested that ERCC6 (this gene encodes a DNA-binding protein that is important in transcription-coupled excision repair [106]) was also a target for let-7c-5p that led to the downregulation of the encoded protein in MCF-7 cells [82].

Interestingly, the ERCC6 mRNA was unaltered, suggesting transcription degradation instead of mRNA degradation. Another study showed the downregulation of let-7c-5p in breast cancer tissues. Furthermore, it was found that let-7c-5p overexpression could inhibit breast cancer cell proliferation [82].

### 5.6. miR-335

miR-335 coding sequence is located on the chromosome 7q32.2 locus and controlled by DNA methylation and was reported to act as an oncogene showing both tumor promoter and suppressor effects depending on the tumor stage and type. BC surpasses tumor invasion and metastasis by downregulating several signal pathways. It also affects the tumor environment and drug sensitivity [85]. Studies show that the overexpression of miR-335 affects cell proliferation, viability, and apoptosis by being a crucial factor in the BRCA1 regulatory network [86]. Interestingly, BRCA1/2 increases the transcription levels of miR-335, which leads to increased cell plasticity and growth [107]. In addition, BRCA1 and EGFR/HER2 can inhibit mRNA maturation, enhancing cell survival and invasiveness [107].

New evidence reports that the downregulation miR-335 in BC suppresses cell metastasis and migration by targeting transcription factor SOX4 and extracellular matrix component tenascin C [108].

The hepatocyte growth factor (HGF)/c-Met pathway contributes to tumor invasion and metastasis and is an essential factor in the progression and prognosis of BC patients [108]. C-Met, being an oncogene, can bind HGF, which induces autophosphorylation of tyrosine residues in c-Met [108]. Studies conducted by Gao et al. in 2014 showed that the forced overexpression of miR-335 revokes HGF-stimulated c-Met phosphorylation and, in consequence, cell migration due to reducing c-Met expression [108]. The same studies indicated that 5-AZA-CdR treatment (DNA methyltransferase inhibitor) significantly increased miR-335 expression, which later influences the HGF/c-Met pathway, and, simultaneously, the level of miR-335 that can play a significant role in breast cancer diagnosis and prognosis and novel strategies for BC therapy [107,108,109]. Research carried out on MDA-MB-231 cells showed that the overexpression of miR-335 could increase the sensitivity of triple-negative breast cancer (BC with negative immunohistochemical results of estrogen receptor, progesterone receptor, and proto-oncogene HER-2) to cisplatin and doxorubicin, which improved the efficacy of chemotherapy [87]. The mechanism involved in increased cell sensitivity still needs to be investigated. It may play an essential role in breast cancer treatment.

### 5.7. miR-126

miR-126, located in the EGFL7 region (a natural negative regulator of vascular elastogenesis), is exclusively expressed in endothelial cells and regulates angiogenic signaling and vascular integrity [88]. Furthermore, it reduces the proliferation and metastasis of tumors by targeting vascular endothelial growth factor (VEGF), which positively regulates vasculogenesis and angiogenesis [88]. It was reported that the expression of miR-126 is downregulated in breast cancer, whereas the VEGF signaling pathway is activated in these cells, which leads to the acceleration of the growth of the tumor [88].

Studies conducted on breast cancer cells MCF7 treated with miR-126 lipofectamine showed evident downregulation of VEGF-A, which is consistent with other studies and shows a negative correlation between upregulation of the VEGF-A expression level and downregulation of the miR-126 expression level. It leads to the conclusion that miR-126 acts as a tumor-suppressive gene and that VEGF-A may be a promising target in breast cancer therapy [110]. One of the drugs used to treat BC is trastuzumab, a monoclonal antibody targeting HER2 receptors, leading to reduced BC cell division, migration, and differentiation [89]. In a study performed by Fu et al., 2020, trastuzumab-resistant SK-BR-3 (SKBR3/TR) cells transfected with miR-126 mimic showed attenuated resistance to trastuzumab while the parental line SK-BR-3 transfected with miR-126 inhibitor showed increased trastuzumab resistance [89]. The same study found that miR-126 directly targets PIK3R2 and is partially involved in the inactivation of the PIK3R2/PI3K/Akt/mTOR signaling pathway responsible for mediated trastuzumab resistance in BC [89]. In conclusion, the overexpression of miR-126 in cells resistant to trastuzumab with inhibition of PIK3R2 and the downstream PIK3R2/PI3K/Akt/mTOR signaling pathway causes a decreased drug resistance [89].

Research conducted by The Affiliated Tumor Hospital of Zhengzhou University showed a correlation between the expression of miR-126 and the regulation of critical metastatic molecule ADAM9 (ADAM metallopeptidase domain 9, a component of cell–cell junctions). Overexpression of this miRNA inhibited breast cancer cell invasion by silencing ADAM9 [90].

Clinical evidence shows that due to increased or decreased expression of specific genes in breast cancer tissue, miR-126 can be used as a biomarker to predict and diagnose breast cancer and therapy response [111].

### 5.8. miR-199a

Recent studies show that overexpression of miR-199a-3p suppresses proliferation, multidrug resistance, migration, and invasion, and it might suppress metastasis progression in breast cancer cells [92]. It also leads to inhibition of PAK4 expression, which has been connected to tumorigenesis and increased cell survival, which is believed to interfere with an aggressive breast cancer phenotype. Targeting the PAK4/MEK/ERK pathway can repress breast cancer progression by inducing G1 phase arrest [92].

Triple-negative breast cancer, accounting for 10–15% of BC, is plagued by significant drug resistance [112]. Studies indicate that in this type of BC, the level of miR-199a-3p is downregulated. It was found that this particular miRNA targets mTOR, which regulates cell proliferation, autophagy, and apoptosis and plays an essential role in cancer cell metabolism [19,112]. Overexpression of miR-199a-3p targets c-Met and mTOR, affecting increased sensitivity to doxorubicin and also leading to G1 phase arrest, resulting in reduced invasion and increased doxorubicin-induced apoptosis in BC cells [112]. The study on MDA-MB-231 cells indicated that miR-199a-3p could downregulate mitochondrial transcription factor A (TFAM) by promoting the sensitivity of BC cells to chemotherapy resistance [91]. In turn, inhibition of TFAM expression could attenuate cisplatin resistance in breast cancer cells and induce apoptotic and proliferative effects [113]. A study regarding the cardiotoxicity of doxorubicin showed that upon doxorubicin exposure, the level of miR-199a expression was upregulated [112]. Considering these findings, miR-199a-3p might be an excellent prognostic and predictive biomarker in breast cancer [114].

### 5.9. miR-101

miR-101 is acknowledged to be a tumor suppressor, and its expression is downregulated in BC [115]. It affects cancer-related processes: proliferation, apoptosis, angiogenesis, drug resistance, invasion, and metastasis. It targets proteasome maturation protein (POMP), stathmin (Stmn1), and DNA (cytosine-5)-methyltransferase 3A (DNMT3A), which suppress the proliferation of BC cells by decreasing the expression levels of Jak2, EYA1, and SOX2 and by reducing levels of VHL, which negatively regulates hypoxia-inducible factor 1-alpha (HIF1alpha), leading to the apoptosis of cancer cells [115]. It was also reported that its high levels in TNBC increase chemotherapeutic sensitivity to paclitaxel by decreasing the level of MCL-1 expression [115].

Brain metastasis is a late event in breast cancer patients. It is a cascade in which metastatic cells detach from the tumor and travel through the bloodstream or lymphatics to arrest into the capillary bed and attach to the brain endothelium, passing through the blood–brain barrier and colonizing the brain [94]. Studies show that overexpression of miR-101-3p reduces the migration of BC cells through the brain endothelium by restraining the COX-2/MMP1 signaling pathway [94].

The experiments conducted in SK-BR-3 and MCF-7 cells showed significant upregulation of an oncogene EZH2, which promotes carcinogenesis and is related to poor prognosis and aggressiveness of breast cancer. Studies have shown that simultaneous induction of miR-101 and treatment with Syn-cal14.1a, a synthetic peptide acquired from Californiconus californicus, suppresses EZH2-induced breast cancer cell migration, invasion, and proliferation and promotes apoptosis of BC cells [116]. Additionally, studies reported that miR-101 played a critical role in the pathological grade in TNM classification in BC cells, making it a promising biomarker [115]. When the miR-101-5p-associated pathways in breast cancer were assessed using RNA-seq, a particular group of genes, HMGB3, ESRP1, GINS1, TPD52, SRPK1, VANGL1, and MAGOHB, were suggested to be associated with a poor prognosis of BC [93].

### 5.10. miR-9

Recent studies have shown the promoting role of miR-9 in breast cancer development [98]. Its upregulation is associated with high malignancy invasive epithelial-to-mesenchymal transition, which enables cells to gain the ability of self-renewal and have the characteristics of stem cells, promoting the production of cancer stem cells (CSCs) which generate an invasive phenotype leading to poor outcome, high tumor stage and histologic grade, poor overall survival, and distant metastasis-free survival [98]. Low miR-9 expression was associated with improved overall survival, smaller tumors, earlier stage, and ER-positive cancers due to the enrichment of estrogen response genes [117]. Furthermore, miR-9 is highly expressed in HER2+ and triple-negative breast cancer and tumors displaying CD44+/CD24- phenotype and E-cadherin loss [98]. Because of the significant engagement of miR-9 in CSCs metabolism, which is considered the origin of tumorigenesis, drug resistance, and development, this miRNA seems a good predictor marker of cancer metastasis and chemoresistance [98]. Studies show that the upregulated expression of miR-9 is induced by MYC and MYCN, which leads to angiogenesis through activation of beta-catenin signaling and elevating the expression of VEGF. It also leads to increased EMT invasiveness and motility by targeting FOXO1 and STRD13, which are also associated with vascular sprouting and promoting tumor metastasis [98]. Another research conducted by Wang et al. indicated that lncTUG1 (taurine-upregulated gene 1) could modulate the susceptibility of BC cells to doxorubicin by regulating the expression of eIF5A2 (eukaryotic translation initiation factor 5A-2) via interacting with miR-9, indicating a novel potential pathway that could be targeted to overcome doxorubicin resistance in BC [95]. Interestingly, NGS results show that miR-9 directly targets HMGA2, EGR1, and IGFBP3, which are closely related to the invasion and metastasis of breast cancer [64].

## 6. miRNA as a Therapy Target

Most miRNAs are found inside the cell but also migrate in body fluids such as blood, urine, saliva, or breast milk. Thus, these short RNA particles are considered diagnostic and therapeutic markers, especially in cancer, neurology, or cardiology [118]. It is noteworthy that miRNA dysregulation is common in many cancer cases as they can act as both tumor suppressors or oncogenes.

miRNA as a therapy target is gaining extensive attention due to its various effects on cancer development. For example, supplementation of miRNA mimics (miR-15a) in prostate cancer cell lines induced apoptosis and blocked cell proliferation [119]. Another study showed that miR-99a reduced breast cancer cell proliferation, invasion, and migration in vitro and in vivo [120]. Numerous studies showed that targeting miRNA with its antagonists might lead to tumor suppression and efficient, personalized cancer therapy [121,122]. Significantly, miRNA-targeted therapy may influence a single gene and whole cellular pathways, which can be particularly beneficial [123]. Specifically, the latest approach in miRNA therapeutics is mainly based on two strategies, i.e., the inhibition of oncogenic miRNAs and, hence, the restoration of the expression of tumor-suppressing genes that they target, or restoring the expression of tumor-suppressing miRNAs and consequently inhibiting the oncogenes that they target. Downregulation of tumor miRNA suppressors leads to the overexpression of their target oncogenes. To restore the expression of tumor-suppressing miRNAs, promising areas are the mimic miRNAs. They are small, chemically modified (2′-O’methoxy) double-stranded RNA molecules that mimic the endogenous mature miRNA molecules [121,124].

Because oncogenic miRNAs are usually upregulated in tumors, their suppression enables tumor suppressors to be active and inhibit tumorigenesis or its progression [121]. For that reason, a few therapeutic strategies based on oncomiR inhibition were created, and one of them is AMOs (anti-mRNA oligonucleotides). AMOs are single-stranded oligonucleotides (17–22 nt long) that prevent mature miRNA interaction with the target gene by complementary binding. As a result, the AMO-miRNA duplex will be cleaved by RNAse-H [121]. By complementary binding with the target mRNA, they exert transcriptional downregulation. Another therapeutic strategy based on oncomiR inhibition is miRNA sponges, which are competitive inhibitors with multiple binding sites for an endogenous miRNA and prevent the interaction between the miRNA and its target mRNA. There is also a strategy based on inhibiting miRNA biogenesis or target interactions via small molecules, like azobenzene [121].

Due to miRNA’s inability to passively diffuse through cell membranes, there is a barrier to miRNA clinical implementation and a need for effective and safe delivery systems development. Nowadays, miRNA delivery systems may be divided into two main categories: non-viral and viral vectors. Non-viral miRNA vectors are based on organic, inorganic, and polymer materials, while viral vectors usually use lentiviruses, retroviruses, or adenoviruses [121]. Other challenges of miRNA therapeutics are associated with its degradation by nucleases, endosomal entrapment, poor target tissue delivery, innate immune reaction activation, and poor binding affinity for complementary sequences [122]. Despite these difficulties, miRNA clinical implications are highly promising [121,122].

### 6.1. The Role of miRNA in Breast Cancer Chemoresistance

Numerous factors, including late diagnosis or resistance to therapeutic agents, may cause therapy failure in cancer therapy. The two basic types of drug resistance, i.e., innate or acquired, constitute a severe challenge in oncology. Recently, both these mechanisms were reported to be associated with miRNAs that modulate drug-resistance-related genes or affect genes related to cell proliferation, cell cycle, DNA damage repair, and apoptosis [125]. Hence, the miRNA-based therapeutic approach seems to provide an interesting and efficient perspective in cancer therapy. Specifically, in breast cancer, several miRNAs were suggested to play a critical role in therapy response, showing a tumor-type-dependent effect. miR-200c, miR-155, and miR-218 were shown to mediate the therapeutic effect of selected drugs, i.e., (i) trastuzumab, (ii) aclitaxel, VP16, doxorubicin, and (iii) cisplatin, respectively [126]. Another study demonstrated 123 miRNAs that were dysregulated in vinorelbine (NVB)-resistant breast cancer cell lines (MDA-MB-231/NVB). A total of 31 of these miRNAs were downregulated, and 92 were upregulated in those cells, suggesting complex regulation [127]. It was also demonstrated that 17 specific miRNAs were involved in oncogenic pathways, including TGFβ, mTOR, Wnt, and MAPK. It is noteworthy that elevated TGFβ signaling and downregulation of miR-200c were also demonstrated in trastuzumab-resistant breast cancer cells while increased miR-200c or the blockade of TNFβ signaling increased trastuzumab sensitivity and inhibited invasiveness of breast cancer cells [128].

Similarly, miR-494 and miR-141 were shown to suppress the progression of breast cancer by repressing β-catenin expression [129,130]. Recently, Yu et al. reported that the miR-17/20 cluster increased tamoxifen sensitivity and attenuated doxorubicin resistance in MCF-7 cells via Akt1 [131]. Another study showed that miR-218, which targets BRCA1, was downregulated in cisplatin-resistant breast cancer cell lines and, interestingly, the restoration of miR-218-sensitized MCF-7 breast cancer cells to this drug [132]. Numerous studies show other miRNAs that are capable of modifying the response of breast cancer cells to different therapeutic agents, including 5-fluorouracil, trastuzumab, lapatinib, cisplatin, fulvestrant, tamoxifen, paclitaxel, doxorubicin, and palbociclib. The most commonly reported BC-related miRNAs (and probably the most critical ones) are presented in Table 1. Recent data suggest that the function of some miRNAs may be involved in the epithelial–mesenchymal transition process that mediates multidrug resistance (MDR) phenotype promotion. A thoroughly revised contribution of miRNAs to individual ABC family transporters was shown elsewhere [133]. Thus, further screening and miRNA profiling in cancer tissues is highly required as it may provide in-depth information regarding critical genes expression regulation. It may be, however, that similarly to wide-genome sequencing that aims to evaluate the role of individual SNPs in genomic DNA, miRNA profiling will not be sufficient to evaluate the risk or monitor disease progression and therapy efficacy. The only possible way seems to be the further assessment of clinical samples that show real mechanistic networks in vivo. Importantly, some clinical trials are being carried out—more than 50 refer to miRNA application in breast cancer [134].

Some translational potential shows the studies that involve a combination of miRNA modulators with anti-cancer chemotherapeutics (specifically, a combination of antagomiRs with therapeutic agents). Alternatively, mimics could be applied that reinforce the function and expression of miRNAs. By affecting the expression of endogenous microRNAs in tumor cells and consequently leading to the modulation of target pathways, they may affect chemotherapy efficacy. However, there are still many difficulties to overcome before we should be able to use miRNAs in the clinical setting, including effective delivery systems, stability, etc.

### 6.2. The Role of miRNA in Breast Cancer Stem Cells

Some recent studies revealed that both cancer stem-like properties and drug resistance were associated with EMT. As mentioned above, miRNAs play a pivotal role in regulating EMT phenotype. As a result, some miRNAs impact cancer stemness and drug resistance [135], which might show some benefits to clinical treatment. Breast cancer stem cells (BCSCs) show self-renewal and differentiation capacities that contribute to the aggressiveness of metastatic lesions, and all these mechanisms can be controlled by regulatory miRNAs [136]. As demonstrated, the expression of microRNAs can be deregulated in BCSCs [137]. Specifically, mir-21, mir-22, mir-29a, and mir-221/222 were shown to increase tumorigenesis, while miR-34a, miR-628, miRNA-140-5p, and miRNA-4319 were reported to decrease metastasis in BCSCs [46,76,138]. The specific pathways targeted by miRNAs are mediated by the key players in cancer development and proliferation, including HIF-1 alpha, PI3K/Akt, and STAT3 signaling, which play critical roles in the prognosis and survival of BCSCs [136].

### 6.3. The Role of miRNA in Cancer Cell Cycle Control

Cell cycle dysregulation is a recognized hallmark of cancer, and its aberrant activation has been related to poor prognosis and drug resistance. Different miRNAs have been described to target genes involved in cell cycle regulation, leading to drug resistance or sensitivity. They were reported not only to target specific pathways but also were shown to be cell cycle step-specific [133].

Several miRNAs have been shown to induce cell cycle arrest due to targeting cyclins. One of them is miR-34a, which was demonstrated to increase resistance to docetaxel (DTX) in luminal BC cells, probably through the inhibition of cyclin D1 (CCND1) and B-cell lymphoma 2 (Bcl-2), inducing G1 arrest and blocking DTX effectiveness as a consequence [139]. miR-93 has also been linked to cell cycle arrest in the G1/S phase. Moreover, some other miRNAs have been shown to modulate drug resistance through targeting CDKs. One of them is miR-29c (targeting directly CDK6), which was downregulated in BC compared to normal tissues [140]. miR-29c overexpression decreased CDK6 level, inducing cell cycle arrest and PTX sensitivity.

Additionally, Citron et al. [141] showed that miR-223 expression levels could predict the effect of CDK4/6 inhibitors and palbociclib (PAB), as well as patients’ prognosis for invasive ductal carcinoma. It was demonstrated that miR-223 was downregulated in luminal and HER2+ BC subtypes. Its low expression was correlated with cell cycle deregulation, poor prognosis, PAB resistance, and low survival in BC patients. Significantly, miRNAs were also shown to affect one of the essential response pathways that are triggered by cancer drugs, i.e., DNA repair pathways, including ATM [142].

### 6.4. miRNAs and Cell Death

Sooner or later, applying specific miRNAs in cancer therapy is supposed to provoke cancer cell death. As demonstrated, it can be caused in a particular manner, also due to miRNA involvement. This makes it again a promising strategy to consider, especially since the miRNA-target gene interactions show numerous effects that directly involve cell death modulators. Some examples are miR-125b, which confers resistance to PTX by suppressing the expression of BAK1 [143], miR-149-5p, which was found to be downregulated in PTX-resistant cells and its overexpression demonstrated to increase BAX expression [144], or miR-663b that confers TAM resistance by indirectly upregulating BAX [145]. Additional miRNAs modulate drug response by regulating the expression of Bcl-2 family members [146]. Moreover, miR-203a-3p and miR-203b-3p have been reported to decrease the antiapoptotic protein Bcl-XL and to be correlated to PTX sensitivity in BC positively regulated by MYC in cell line models of PTX-responsive BC [147].

Interestingly, miR-100 was found to be downregulated in BC cell lines with acquired resistance to CIS. In turn, overexpression of miR-100 showed increased sensitivity to CIS due to modulation of the HCLS1-associated protein X-1(HAX-1), an inhibitor of mitochondrial apoptosis that maintains mitochondrial membrane potential in cancer cells [148]. miR-944 inhibitors facilitated CIS-induced loss of mitochondrial membrane potential in resistant models, resulting in intrinsic apoptosis via targeting Bcl-2 interacting protein 3 (BNIP3) [148].

Similarly, miRNAs control critical mediators of apoptosis [149] and autophagy [150] at different levels, including PI3K/Akt/mTOR, ATGs, and LC3 [150]. Primary reports showed some specific miRNAs that affected STAT3 and ATG12 targets [151], while further studies demonstrated broader roles of autophagy-related microRNAs in cancer cells [152], showing numerous miRNAs acting at the levels of induction, nucleation, expansion, fusion, degradation, and recycling. With so many miRNA particles and the dynamics of autophagy, it is difficult to show a specific pattern that would apply to any specific cancer type. However, as miRNAs target specific genes, monitoring their expression during promoting (e.g., rapamycin, everolimus) or inhibiting autophagy (e.g., chloroquine, hydroxychloroquine) may reflect metabolic alterations that accompany different stages of therapy. Thus, we can evaluate the therapy efficacy and indicate molecular targets for more efficient therapeutic strategies. However, the pool of the genes that effectively affect pathways associated with autophagy, i.e., energy, growth, starvation response, etc., can be modulated by over 250 miRNA-target gene interactions in different cellular stress response mechanisms [151], which may make the whole idea more complex.

## 7. Tools in miRNA-Based Therapy Adjustment

Modulation of gene expression seems to be one of the best ways to control cell metabolism against all odds, including mutations or epigenetic factors. Overcoming these obstacles enables controlling of the phenotype, i.e., metabolism, structure, enzyme activity, substrate affinity, and protein stability. Altogether, it provides quantity and quality of cell metabolism that eventually affects the quality and the length of human life. However, using miRNA or targeting this non-coding RNA requires first identification of specific interactions as well as tissue-type and personalized profiling. This can be achieved by RNA-seq or spatial transcriptomics that deliver information on the whole transcriptome. Another step is to find a pattern—an assessment of association analysis that enables distinguishing health and disease. This approach can be obtained using different data systems, e.g., TargetScanHuman 8.0 [59], that can predict biological targets of miRNAs by searching for the presence of conserved 8mer, 7mer, and 6mer sites that match the seed region of each miRNA. The results demonstrate predictions with adjustable high and low conserved sites ranked based on the predicted efficacy of targeting.

Another option is to use Xena Browser [153] or Gepia2 (http://gepia2.cancer-pku.cn/#index, accessed on 9 February 2023) [154] to identify any alterations in the levels of selected miRNAs in different cancer tissues. Similarly, another system, On-coLnc (http://www.oncolnc.org/, accessed on 27 September 2023) [155], can link TCGA survival data to mRNA, miRNA, or lncRNA expression levels. Altogether, we have some sophisticated and advanced tools that enable prediction and assessment of the miRNA profiles. The main goal would be to find a characteristic and unique profile of the oligonucleotides that show significant association with clinical characteristics and patient outcomes.

## 8. Conclusions: Challenges in miRNA Modulation Approach

Since miRNAs control the expression of numerous target genes, it is unsurprising that they play critical roles in regulating cell metabolism. Thus, they have recently become the primary candidates for markers in cell homeostasis imbalance detection, disease diagnostics, and prognostics. We still study the miRNA-target gene interactions’ role, mechanism, and specificity. Importantly, it was demonstrated that these short oligonucleotides showed significant stability in the extracellular space and were reported to mediate functional communication between cells. It is mainly associated with their ability to transfer between cells via extracellular vesicles (EVs) or other cell-free miRNA carriers [156,157]. This, in turn, raises the question about the tissue specificity of their expression/localization. Another critical challenge is that miRNAs target multiple genes with different efficacies that may not show specific effects after target miRNA modulation. The sequence complementarity of endogenous miRNAs ranges between 20 and 90% [158]. Surprisingly, in specific conditions (e.g., starvation), some miRNAs can upregulate the expression of target genes or lead to induction of the immune system and provoke severe adverse effects (e.g., miR-34a mimic and targeting miR-122 evaluation was discontinued after phase I and phase II, respectively) [158].

A single miRNA can target many mRNAs, and a single mRNA can be targeted by many miRNAs (many in this case means at least hundreds), which makes identification of precise interactions or using a specific miRNA as a target extremely difficult. Theoretically, using in silico algorithms, we can predict the miRNA–mRNA binding strength. However, the biological effect will depend on multiple factors, such as the level/stability of selected miRNA, the level of other miRNAs that target the same mRNA, the level of mRNA/target gene expression, and the availability of AGO2. Additionally, the complexity level significantly increases due to the earlier-mentioned ability of miRNAs to be transferred between different cells.

However, more questions refer not only to the specificity aspect but also to safety and side effects issues, formulation and bioavailability problems, and efficacy challenges. These aspects are also important when miRNAs are combined with certain drugs, which may lead to some metabolic interactions [159]. However, the issues appear also at the delivery step. It results from the fact that most miRNA modulators are negatively charged, which leads to nonspecific binding to blood proteins and decreases urinary clearance [160]. On the other hand, oligonucleotides that lack a charge weakly bind to plasma proteins and exhibit a rapid clearance either due to metabolism in the blood or excretion via urine, leading to a lower tissue uptake [160,161]. Although numerous clinical trials using miRNAs are being carried out, they have yet to show efficient solutions for the above-mentioned reasons. From the diagnostic perspective, miRNAs also show some limitations mainly associated with the overlapping activities and effects of selected miRNAs, which show limited specificity in diagnosing a specific cancer type [162]. Thus, we should instead focus on profiling miRNA levels and creating some diagnostic panels that could be used to improve the classification system and therapy planning.

Significantly, novel, personalized, and precise medicine is based on the identification of specific biomarkers but also on robust and versatile analytical technologies that improve patient outcomes [162]. The group of methods capable of identifying miRNAs and meeting the high sensitivity criteria includes quantitative reverse transcriptase PCR, digital PCR, microarray, or next-generation sequencing modified to miRNA-seq. All these methods have some limitations (including technical, standardization, reference controls, etc.) that were thoroughly discussed elsewhere [163]. Altogether, miRNA biosynthesis control and extracellular trafficking pathways constitute a challenging aspect of miRNA-based therapeutic or diagnostic strategies, significantly since they can be affected by environmental and uncontrollable factors (such as smoking, diet, circadian cycles, etc.) [164].

## Figures and Tables

**Table 1:** The list of critical miRNAs associated with breast cancer (according to [48], modified) reported as good diagnostic or therapeutic candidates. The candidates were selected based on the latest reports indicating the role of miRNA in breast cancer. Consequently, a broad analysis of selected miRNA targets suggested some good candidate markers based on the global data at TargetScanHuman 8.0 [59].

miRNA	Regulation in Breast Cancer Cells	Source and Detection Method	Target	Target Effects/Action	Metabolic Consequences
miR-21	Upregulated	Serum, qRT-PCR [60]	PTEN [44,58]	Downregulation of PTEN expression [44,58,61]	Drug resistance to doxorubicin in HER2- BC cells [61]
				* miR-21 inhibition induces PTEN expression [62]	* Restored trastuzumab sensitivity in the resistant BC xenografts in vivo [62]
			PTEN/Akt [63]	Downregulation of PTEN expression and Akt activation [63]	Induction of EMT and gemcitabine resistance [63]
			PI3K/Akt, MEK/ERK [58]	Activation PI3K/Akt and MEK/ERK signaling pathways [58]	Development of MDR [58]
			TPM1, TGF-β [64]	Repression of expression TPM1 [65,66]	Increased BC cells proliferation, migration, invasion, survival, and EMT [64]
			Mesenchymal cell markers (N-cadherin, Vimentin, α-SMA) [67]	Activation of mesenchymal cell markers [67]	Re-expression of miR-21 is responsible for migration and invasion by activating the EMT process in MCF7 cells [67]
			Epithelial cell marker (E-cadherin) [67]	Inhibition of epithelial cell marker [67]	
miR-106a	Upregulated	Serum, qRT-PCR [68]	Bcl-2,ABCG2, BAX, P53, RUNX3 [69]	Upregulation of Bcl-2 protein and multidrug transporter ABCG2. Downregulation of BAX protein and genes products: P53, RUNX3 [69]	Promotes BC cells proliferation and invasion [69]
				* Inhibition of miR-106a downregulates the expression of Bcl-2, ABCG2 and upregulates the BAX, P53, RUNX3 expression [69]	
			RAF-1 [68]	Decreases RAF-1 levels and RAF-1 is a part of MAPK/ERK signaling pathway [68]	Possibly induces proliferation and decreases apoptosis in BC cells through regulation of the MAPK/ERK signaling pathway, which controls gene expression [68]
			ZBTB4 [70,71,72]	Negative regulation of ZBTB4 gene, which functions as a tumor suppressor gene [70,71,72]	* Restoration of ZBTB4 suppress Sp1, Sp3, Sp4 expression resulting in inhibition of BC cells proliferation, invasion [70,71,72]
miR-155	Upregulated	Serum, qRT-PCR [73]	TERF1 [74]	Reduction in the shelterin component TRF1 expression. TRF1 regulates telomere length and suppresses DNA breakage [74]	Antagonization of telomere integrity in BC cells and increased genomic instability [74]
			SOCS1 [75]	Repression of SOCS1 (negative feedback regulator of JAK/STAT signaling) [75]	Constitutive activation of STAT3 in BC cells, promotion of cell proliferation and colony formation [75]
			C/EBPβ [76,77]	Loss of CCAAT-enhancer binding protein beta (C/EBPβ) [76,77]	Modification of TGF-β response; from growth inhibition to EMT, invasion, and metastasis in BC. Promotion of BC progression [76,77]
mir-141	Downregulated	Tissue, qRT-PCR, Microarray [78]	ANP32E [78]	Regulation of ANP32E (positive regulator of tumor growth and metastasis) [78,79]	ANP32E induces tumorigenesis of BC by upregulating E2F1 and promoting the G1/S transition [79]
				** Overexpression of miR-141 downregulated ANP32E expression [78]	** Inhibition of BC cells proliferation, migration, and invasion [78]
			SIP1 [80]	Regulation of EMT [80]	EMT plays a crucial role in early tumor metastasis and SIP1 is a promoter of cancer progression [80]
let-7c	Downregulated	Serum, qRT-PCR [81]	ERCC6 [82]	Upregulation of ERCC6 [82]	Intensified cancer growth ability and lower rate of apoptosis; DNA damage accumulation [82]
			BCL2, BAX [83]	** Overexpression of let-7c decreases level of Bcl-2 and increases the level of BAX, TP53, PTEN [83]	** Promotion of apoptotic cell death, suppression of cancer progression [83]
			ERα and Wnt signaling [84]	** Overexpression of let-7c inhibits estrogen induction in ERα and Wnt signaling [84]	** Inhibition of BCSCs self-renew and suppresses tumor formation [84]
miR-335	Downregulated	Serum, qRT-PCR [85]	BRCA1 [86,87]	Downregulation of BRCA1 [86]	Accelerated tumor growth, genomic instability, BC progression [86]
				** Overexpression of miR-335 upregulates the level of BRCA1 [86,87]	** Decreased cell viability and increased apoptosis [86,87]
miR-126	Downregulated	Tissue, qRT-PCR [88]	VEGFA [88], PIK3R2 [89]	Inactivation of the PIK3R2/PI3K/Akt/mTOR signaling pathway [89]	Vasculogenesis, angiogenesis resulting in tumor growth [88] Resistance to trastuzumab [89] in SKBR3 and BT747 cell lines
			ADAM9 [90]	** Upregulation of miR-126 is silencing ADAM9 gene [90]	** Inhibition of BC cells invasion and metastasis [90]
miR-199a	Downregulated	Tissue, qRT-PCR [91]	PAK4/MEK/ERK signaling pathway [92]	Regulation of PAK4/MEK/ERK signaling pathway [92]	PAK4 activates the ERK pathway, and MEK/ERK pathway plays a part in PAK4-induced cell growth regulation [92]
				** MiR-199a/b-3p downregulates PAK4 expression and PAK4/MEK/ERK signaling pathway [92]	** Suppression of BC cells migration and invasion [92]
miR-101	Downregulated	Tissue, qRT-PCR [93]	COX-2/MMP1 signaling pathway [94]	Upregulation of COX-2/MMP1 signaling pathway [94]	Promotes transmigration of metastatic BC cells through the brain endothelium [94]
				** Restoring miR-101-3p in BC cells reduces COX-2/MMP1 expression [94]	** Reduction in transmigratory ability [94]
miR-9	Upregulated	Cell culture, qRT-PCR [95]	FOXO1 [96]	Downregulation of FOXO1 expression [96]	Promotion of proliferation, migration, and invasion of BC cells [96]
			STARD13 [97]	Repression of STARD13 [97]	Upon stimulation of PDGFRβ signaling, miR-9 could promote the formation of vascular-like structures of TNBC [97]
			E-cadherin [96,98]	E-cadherin downregulation [98,99]	Increased tumor angiogenesis [99] Primes BC cells to EMT and invasion [98]

* refers to report showing effects of miR inhibition. ** refers to report showing effects of miR activation.

## Abbreviations

ABCG2	adenosine triphosphate binding cassette subfamily G member 2
ADAM9	disintegrin and metalloproteinase domain-containing protein 9
ADC	antibody-drug conjugate
Akt	protein kinase B
AMOs	anti-messenger ribonucleic acid oligonucleotides
ANP32E	acidic nuclear phosphoprotein 32 family member E
ATG12	autophagy-related gene 12
ATM	ataxia telangiectasia mutated kinase
BAK1	Bcl-2 homologous antagonist killer 1
BAX	Bcl-2 associated X protein, apoptosis regulator
BC	breast cancer
Bcl-2	B-cell lymphoma 2
BCSCs	breast cancer stem cells
BNIP3	BCL2 interacting protein 3
BRCA1/2	breast cancer gene 1/2
CCND1	cyclin D1
CDKs	cyclin-dependent kinases
cDNA	complementary deoxyribonucleic acid
CIS	cisplatin
COX-2	cyclooxygenase 2
CSCs	cancer stem cells
Dicer	endoribonuclease Dicer
DNA	deoxyribonucleic acid
DNMT3A	deoxyribonucleic acid methyltransferase 3 alpha
DOX	doxorubicin
DTX	docetaxel
c-Met	mesenchymal-epithelial transition factor
EGFL7	epidermal growth factor-like protein 7
EMT	epithelial-to-mesenchymal transition
ER	estrogen receptor
ERBB2	erythroblastic oncogene B2
ERCC6	deoxyribonucleic acid excision repair protein
ERKs	extracellular signal-regulated kinases
FDA	Food and Drug Administration
FOXO1	forkhead box protein O1
GDP	guanosine diphosphate
GEFs	guanine–nucleotide exchange factors
GTP	guanosine triphosphate
HAX-1	HCLS1-associated protein X-1
HER2	human epidermal growth factor receptor 2
HGF	hepatocyte growth factor
IGFBP3	insulin-like growth factor binding protein 3
JAK/STAT	janus kinase/signal transducer and activator of transcription
MAPKs	mitogen-activated protein kinases
MDR	multidrug resistance
MMP1	matrix metallopeptidase 1
mRNA	messenger ribonucleic acid
miRNA	micro-ribonucleic acid
mTOR	mammalian target of rapamycin
NF-κB	nuclear factor kappa-light-chain-enhancer of activated B cells
NVB	vinorelbine
P53	tumor protein p53
PAB	palbociclib
PARP	poly (ADP-ribose) polymerase
PAK4	serine/threonine-protein kinase
PDCD4	programmed cell death protein 4
PI3K	phosphoinositide 3-kinase
PIK3R2	phosphoinositide 3-kinase regulatory subunit 2
Pol II	ribonucleic acid polymerase II
POMP	proteasome maturation protein
PR	progesterone receptor
pri-miRNA	primary micro-ribonucleic acid
pre-miRNA	precursor micro-ribonucleic acid
PTEN	phosphatase and tensin homolog
PTX	paclitaxel
RAF	rapidly accelerated fibrosarcoma
RISC	ribonucleic acid-induced silencing complex
RNA	ribonucleic acid
RNase	ribonuclease
RTKs	receptor tyrosine kinases
RUNX3	runt-related transcription factor 3
ssRNAs	single-stranded ribonucleic acids
STAT3	signal transducer and activator of transcription 3
TFAM	transcription factor A, mitochondrial
TGF-β	transforming growth factor-beta
TKI	tyrosine kinase inhibitors
TNM	tumor: node, metastasis
TPM1	tropomyosin 1
VEGF	vascular endothelial growth factor
WHO	World Health Organization
RT-qPCR	quantitative reverse transcription polymerase chain reaction
3′UTR	three prime untranslated region
5′UTR	five prime untranslated region
